# Anti-Bacterial Effects of Essential Oils against Uropathogenic Bacteria

**DOI:** 10.3390/antibiotics9060358

**Published:** 2020-06-25

**Authors:** Maria Loose, Emmelie Pilger, Florian Wagenlehner

**Affiliations:** Clinic for Urology, Paediatric Urology and Andrology, Justus-Liebig University of Giessen, 35392 Giessen, Germany; Emmelie.Pilger@med.uni-giessen.de (E.P.); Florian.Wagenlehner@chiru.med.uni-giessen.de (F.W.)

**Keywords:** essential oils, phytotherapy, urinary tract infection, cystitis, antimicrobial resistance

## Abstract

Given the increasing antimicrobial resistance in urinary tract infections (UTI), alternative strategies need to be investigated. Determination of minimal inhibitory and bactericidal concentrations of essential oils from cajeput, lemongrass, tea tree, and thyme in artificial urine, revealed bactericidal activity of all four tested essential oils against seven uropathogenic species with values ranging between 0.78–50 mg/mL. Tea tree and thyme essential oils were more efficient than lemongrass and cajeput. In addition, antibiotic-resistant strains showed similar susceptibility as antibiotic-sensitive strains, suggesting no cross-resistance between antibiotics and these essential oils. Checkerboard assays revealed a synergistic activity of the combination of thyme and tea tree. Furthermore, the combination with thyme and tea tree essential oils increased the activity of fosfomycin and pivmecillinam, but not nitrofurantoin, against *Escherichia coli*. This study provides a basis for further investigation of the potential of thyme and tea tree oil as an alternative or additional treatment of UTI.

## 1. Introduction

Uncomplicated urinary tract infections (UTI), which are caused predominantly by *Escherichia coli* but also by *Staphylococcus saprophyticus*, *Proteus mirabilis*, and *Klebsiella* spp. and other Enterobacteriaceae, are amongst the most common reasons for medical consultation [1]. For the treatment of mild and moderate uncomplicated UTI, oral antibiotics are preferred [2]. However, due to the increasing rates of resistance of uropathogens against cotrimoxazole, fluoroquinolones, and β-lactams, these classical oral antibiotics cannot be used for empiric therapy any more in many geographic regions [3,4]. To counteract the increasing resistance rates, alternative treatment options, such as phytotherapeutic approaches, need to be investigated.

Essential oils, also known as volatile oils, are products of the secondary metabolism of aromatic plants. Various essential oils have been reviewed to possess different biological properties such as anti-inflammatory, sedative, digestive, antimicrobial, antiviral, or antioxidant activities [5]. Since the middle ages, essential oils have been widely used for bactericidal, virucidal, fungicidal, parasitical, insecticidal, medicinal, and cosmetic applications, especially nowadays in pharmaceutical, sanitary, cosmetic, agricultural, and food industries [5]. In recent years, direct killing as well as sensitizing activities against microbes have been reported for essential oils [5,6,7]. Essential oils of the genus *Origanum* L. showed promising antibacterial effects against uropathogenic *E. coli* including even multi-drug resistant strains [8,9,10]. A recent randomized study in patients with lower uncomplicated UTI showed that non-antibiotic herbal therapy was non-inferior to antibiotic therapy in the treatment of the acute phase of UTI [11]. In addition, essential oils of *Pelargonium graveolens* and *Coriandrum sativum* were shown to potentiate the effectivity of ciprofloxacin and gentamycin, respectively, against selected uropathogens [12,13].

In this study, the antibacterial activity of purchasable essential oils from *Cymbopogon flexuosus* (Nees ex Steud.) W. Watson (lemongrass), *Melaleuca alternifolia* (Maiden & Betche) Cheel (tea tree), *M. leucadendron L. var cajaputi* (cajeput), and *Thymus vulgaris* L. c.t. linalool (thyme) was tested against different uropathogenic species in artificial urine. By further checkerboard assays, potential synergistic activity combining different essential oils together or combining essential oils with antibiotics were determined.

## 2. Results

### 2.1. Chemical Composition of the Essential Oils

The essential oils of cajeput, lemongrass, tea tree, and thyme obtained by PRIMAVERA LIFE GmbH. The oils were extracted by steam distillation by the manufacturer. According to the manufacturer’s certificate of analysis, the gas chromatography/mass spectrometry (GC/MS) analysis identified 22–31 components, which accounted for 93–100% of the total composition. The main detected components were 1,8-cineole (64.13%) and α-terpineol (11.43%) for cajeput essential oil and neral (28.49–33.88%) and geranial (36.45–43.38%) for lemongrass essential oil. Terpinen-4-ol (40.39%) and α/γ-terpinene (9.49/20.20%) were the major components of the tea tree essential oil, whereas linalool (44.56–57.28%) dominated in thyme essential oil, followed by terpinen-4-ol (7.57–10.51%) (Table 1).

### 2.2. Minimum Inhibitory and Bactericidal Concentration (MIC/MBC) Determinations

Seven different species of uropathogenic bacteria, including antibiotic-resistant strains, were tested for determination of inhibitory and bactericidal activity of the essential oils. MIC/MBC values almost do not differ for all tested strains for the different essential oils (Table 2). Thus, all of the essential oils tested show more bactericidal than bacteriostatic activity. For *E. coli*, tea tree and thyme showed the best activity with MIC values ranging between 0.78–3.13 mg/mL for all eleven strains tested. In contrast, MIC/MBC values for lemongrass and cajeput showed intraspecies differences with values ranging from 1.56 up to 50 mg/mL. Since strains without known antibiotic resistance also showed higher MIC values (Table 2), this reduced susceptibility cannot be linked to existing antibiotic resistance. For *K. pneumoniae* and *E. cloacae*, similar results with intraspecies differences, especially for lemongrass and cajeput, were obtained. For the tested *S. saprophyticus* strain, all four tested essential oils showed similar bactericidal activity with a range of 0.78–1.56 mg/mL. *E. faecalis* was more susceptible against lemongrass compared to the other three essential oils, whereas *P. mirabilis* was most susceptible against tea tree oil. *P. aeruginosa* was the least susceptible species against all four essential oils tested, with MIC values ranging between 12.5–50 mg/mL.

### 2.3. Checkerboard Assays

Furthermore, we were interested in whether the seen bactericidal activity of the essential oils could be increased by combining two of them. Therefore, we tested for synergistic activity using checkerboard assays against antibiotic-sensitive *E. coli* strains as the major causative agent for uncomplicated cystitis. Strains were selected according to their sequence types to represent four of the most prevalent sequence types of cystitis causing *E. coli*—131 (*E. coli* CHD94), 95 (*E. coli* UTI89), 73 (*E. coli* CHD3), and 69 (*E. coli* CHD22) [14]. The combination of thyme and tea tree resulted in an additive 8-fold reduction of the MIC and MBC values of both essential oils compared to the substances alone (Table 2). To further test whether essential oils could be beneficial for UTI treatment, we examined the effects of the combination of thyme and tea tree with three of the guideline-recommended antibiotics for the treatment of uncomplicated lower UTIs—fosfomycin, nitrofurantoin, and pivmecillinam [2]. The combination of nitrofurantoin with both oils only had a mild beneficial effect with reduction of MIC/MBC values up to 2-fold (Table 3). The addition of tea tree oil or thyme reduced fosfomycin MIC and MBC values about 2–4-fold, which was additive based on fractional inhibitory/bactericidal concentrations (FIC/FBC) indices. In addition, for pivmecillinam, additive reduction of MIC and MBC values of 2–8-fold could be observed (Table 3).

## 3. Discussion

Essential oils of cajeput, lemongrass, tea tree, and thyme exhibited antibacterial activities against all tested uropathogenic bacterial strains, whereby the susceptibility of individual species differed. Here, *P. aeruginosa* showed the least susceptibility, which corresponds to previous studies [7,15]. An external membrane, which is particularly impermeable to essential oil molecules in combination with efflux mechanisms, might be responsible for the high resistance of *P. aeruginosa* to essential oil [15]. Overall, essential oils from thyme and tea tree were more efficient against the tested bacteria than lemongrass and cajeput. Assuming that the main components play a major role in antibacterial activity, it can be postulated that linalool and terpinen-4-ol are more effective against uropathogenic bacteria than 1,8-cineole and neral/geranial. Disruption of the bacterial cell wall and membrane integrity is shown as the antibacterial mechanism for all of them [16,17,18,19]. However, their exact mechanism of action is still unknown. Comparative studies on the antibacterial activity of the individual terpenes are rare. One study showing that linalool type essential oils of *Litsea cubeba* were more effective than 1,8-cineole type essential oils against various bacterial species including *E. coli* [20], supports our hypothesis. Nevertheless, further investigations are still needed. *E. coli* strains harboring different antibiotic resistances were as susceptible to tea tree and thyme essential oils as the antibiotic-sensitive strains, suggesting no existing cross-resistance. In contrast, especially lemongrass showed large intraspecies differences in MIC/MBC values. However, since increased MIC values were obtained for both antibiotic-resistant and sensitive strains, a connection between antibiotic resistance and essential oil resistance can be excluded. Intraspecies differences are more likely responsible for these variations. *E. coli* strains can be classified according to their sequence types that can be further assigned to different phylogenetic groups (A, B1, B2, D, E and F). The phylogenetic groups differ in their ecological niches and characteristics, such as their ability to exploit different sugar sources, their growth rate, and their antibiotic-resistance profile, due to a different genetic background and phenotypic manifestation [21]. Differences in membrane composition or expression of efflux machineries between subtypes could be responsible for varying susceptibilities against the antimicrobial components of the lemongrass essential oil. Checkerboard analysis revealed synergistic activity for tea tree and thyme essential oils, showing an increased antimicrobial activity of a combination of both. Furthermore, combination of essential oils with antibiotic commonly used for the treatment of uncomplicated cystitis, revealed additive effects for the combination of fosfomycin or pivmecillinam with tea tree or thyme. Hence, additional essential oil treatment could increase the effectivity of the antibiotic therapy. Furthermore, anti-inflammatory effects described for essential oils [22] could help to reduce the infection-caused inflammation and the associated pain accompanying UTI. Current application recommendation of essential oils for cystitis treatment includes hip baths (i.e., sitting 10–15 min in essential oils solved in warm water) or topical application by warm compresses or massages on the lower back, tights, and pelvis. However, studies showing to what extent the bioactive compounds of the oils reach the bladder are missing. Thus, there are no evidences that concentrations shown here to act bactericidal can be achieved in the bladder by these applications.

Essential oils are complex mixtures containing 20–60 components, which are characterized by one to three major ingredients at high concentrations. The used essential oil of thyme contains about 50% linalool, whereas Terpinen-4-ol (~40%) and α-/γ-terpinene (~10/~20%) are the major components of the tea tree oil (Table 1). Previous reports suggest that these components make up a significant part of the antimicrobial activity of the essential oils. Linalool, as well as Terpinen-4-ol alone, were shown to act bactericidal and fungicidal [18,23,24,25]. Furthermore, anti-inflammatory effects were described for these substances [26,27,28]. Studies in rats showed that 55% of orally administrated linalool was excreted in the urine as glucuronic acid conjugate [29,30], suggesting a possible oral dosage as UTI treatment. Oral intake could allow targeted concentrations to be reached in the urine by dose optimization. Nevertheless, investigations into whether the glucuronic acid conjugation influences the bactericidal activity have to be done first. Furthermore, pharmacokinetic and -dynamic determinations in humans is urgently needed to prove their safety and activity.

The bactericidal activities against uropathogens and the additive effects on antibiotic treatment of tea tree and thyme essential oils, in combination with their known anti-inflammatory impact, suggest a beneficial usage of these essential oils, alone or in combination with antibiotics, for treatment of uncomplicated UTIs. Since the antimicrobial activity of the essential oils seems not to be influenced by antibiotic resistances, they could be a promising alternative for treatment of antibiotic-resistant strains. Nevertheless, since concentrations of essential oils reaching the bladder by hip bath or massages are not known, the possibility of using isolated main components such as linalool and terpinen-4-ol as oral therapy should urgently be further investigated in order to enable a more targeted application.

## 4. Materials and Methods

### 4.1. Bacterial Strains and Materials

*E. coli* ATCC25922, *K. pneumoniae* BAA1705, *K. pneumoniae* BAA2146, and *P. aeruginosa* ATCC27853 were obtained from ATCC (Manassas, VA, USA). *E. coli* UTI89 was obtained from the DSMZ (Braunschweig, Germany). All other strains used in this study were clinical isolates from urine samples. Commercial essential oils were obtained from PRIMAVERA LIFE GmbH (Oy-Mittelberg, Germany). Fosfomycin (TCI, Eschborn, Germany), nitrofurantoin (Cayman Chemical, Ann Arbor, Michigan, USA), and pivmecillinam (Sigma Aldrich, Darmstadt, Germany) were used for combination testing.

### 4.2. Determination of Minimal Inhibitory and Bactericidal Concentrations (MIC/MBC)

MIC/MBC determinations were performed according to the CLSI- and EUCAST-Standards. Broth microdilution assay was used for determination of the MIC in artificial urine (AU—contains [g/L]: CaCl_2_—0.49, MgCl_2_·6H_2_O—0.65, NaCl_2_—4.6, Na_2_SO4—2.3, Na_2_ citrate · 2H_2_O—0.65, Na_2_C_2_O_4_—0.02, KH_2_PO_4_—2.8, KCl—1.6, NH_4_Cl—1.0, Urea—25.0, gelatine—5.0, and tryptone soya broth—10.0; pH 6.1 [31]). Essential oils were first diluted in absolute ethanol and afterwards added to AU in the 96-well plate with a starting concentration of approximately 50 mg/mL (*v*/*v*). Afterwards, 2-fold serial dilutions in AU were performed. The MIC was defined as the lowest concentration inhibiting visible growth (OD600 < 0.1) after incubation at 37 °C for 20 ± 2 h. For determination of MBCs, in a second step, 3 µL of each well of the cultured plate were transferred onto CAMHB agar supplemented with 5% blood (Oxoid) using a one-time inoculator (Dr. Brinkmann Floramed, Nürtingen, Germany). The plates were incubated overnight at 37 °C. The number of colonies subsequently grown was used to determine the bactericidal endpoint. MBC was defined as a > 99.9% (>3-log) reduction of the initially inoculated colony counts. MIC/MBC determinations were performed thrice.

### 4.3. Checkerboard Assays

A two-dimensional, two-agent broth microdilution checkerboard titration method was used to study the interaction between essential oils and antibiotics [32]. Serial 2-fold dilutions were prepared in artificial urine. One tested substance was added to a 96-well plate along the ordinate, the combination partner along the abscissa. The tested concentration for the thyme and tea tree essential oils ranged from 6.25 to 0.097 mg/mL. For fosfomycin, nitrofurantoin, and pivmecillinam, the concentrations tested were 64–2 µg/mL, 16–0.5 µg/mL, and 8–0.25 µg/mL, respectively. Layouts of checkerboard assays plates included testing both substances alone and in combination as well as a positive (without added antibiotic or essential oil) and negative (media without bacteria) control. After 20 ± 2 h of incubation at 37 °C, the MIC and MBC were determined as described above. Checkerboard determinations were performed in triplicates.

Interactions were then evaluated using the fractional inhibitory/bactericidal concentrations indices (FICI/FBCI) calculated as the sum of the FIC or FBC as follows:

FICI = FICA + FICB

FIC-MIC of the substance in combination/MIC of the substance alone.

Correlation between FICI/FBCI and the effect of the combination according to EUCAST definition: synergy ≤ 0.5, additive > 0.5–1, indifference > 1 to < 4, and antagonism ≥ 4.

### 4.4. Statistical Analysis

Differences between two groups were assessed using the Student’s t-test. Statistical calculations were performed using Microsoft Excel 2016.

## Figures and Tables

**Table 1:** Country of origin, type of cultivation and production, used batch numbers, and chemical composition * of the used essential oils according to the certificates of analysis of the manufacturer PRIMAVERA LIFE GmbH.

Cajeput		Lemongrass		Tea Tree		Thyme	
Cambodia/wild harvesting		Nepal/organic cultivation		Australia/organic cultivation		Spain and France/organic cultivation	
Steam distillation of the leaves and tops of *Melaleuca leucadendron L. var cajaputi*		Steam distillation of freshly cut and slightly dried grass of *Cymbopogon flexuosus*		Steam distillation of the leaves of *Melaleuca alternifolia*		Steam distillation of blooming herbs from *Thymus vulgaris* c.t. linalool	
Batch 00284C27 and 00012A27		Batch: 00536M26 and 00290E27		Batch: 00040D27		Batch: 00689F25 and 00146J26	
Component	%	Component	%	Component	%	Component	%
α-pinene	1.76	camphene	1.12–2.71	α-thujene	0.91	α-pinene	2.22–3.35
benzaldehyde	0.13	6-methyl-5-heptene-2-one	1.46–2.95	α-pinene	2.52	camphene	0.54–0.95
β-pinene	1.27	limonene + c-β-ocimene	0.93–2.27	sabinene	0.28	1-octene-3-ol + sabinene	0.82–1.12
myrcene	1.23	exo-isocitral	0.44–0.66	β-pinene	0.71	β-pinene	0.54–0.66
α-terpinene	0.23	citronellal	0.48–0.54	β-myrcene	0.84	β-myrcene	4.91–8.86
*p*-cymene	0.37	photocitral A	<0.01–0.34	α-phellandrene	0.44	α-phellandrene	0.27–0.52
limonene	5.26	isoneral	1.13–1.80	α-terpinene	9.49	α-terpinene	2.25–4.04
1,8-cineol	64.13	isogeranial	1.86–2.81	*p*-cymene	2.63	*p*-cymene	1.24–3.67
γ-terpinene	0.74	1-decanal	0.15–0.24	limonene	0.85	limonene	1.73–3.04
linalool	2.69	citronellol + nerol	0.22–0.59	β-phellandrene	0.85	β-phellandrene	0.46–0.53
δ-terpineol	0.24	neral	28.49–33.88	1,8-cineol	2.08	1.8-cineol	0.56–0.59
terpinen-4-ol	0.78	geraniol	0.83–7.82	γ-terpinene	20.26	γ-terpinene	5.14–6.87
α-terpineol	11.43	geranial	36.45–43.38	ledene + bicyclogermacrene	0.79	c-sabinene hydrate + c-linalool oxide	0.96–1.18
geraniol	0.26	geranyl acetate	0.20–4.03	linalool	0.06	terpinolene + t-linolool oxid	1.19–2.06
eugenol	0.04	β-carophyllene	0.66–1.85	c-*p*-Menth-2-en-1-ol	0.27	linalool	44.56–57.28
α-ylangene	0.18	isoeugenol	0.13–0.14	t-*p*-Menth-2-en-1-ol	0.20	hotrienol	0.64–1.44
β-caryophyllene	1.21	γ-cadinene	1.08–1.26	Terpinen-4-ol	40.39	c-*p*-Menth-2-en-1-ol	0.18–0.24
α-humulene	0.80	δ-cadinene	0.27–0.31	α-terpineol	2.81	t-*p*-Menth-2-en-1-ol	0.16–0.18
β-selinene	1.29	caryophyllene oxide	0.46–0.51	methyleugenol	0.05	camphor	0.51–0.77
α-selinene	0.88	4-nonanone	<0.01–1.37	t-β-caryophyllene	0.42	borneol	1.10–1.61
guaiol	0.66	linalool	1.19–1.39	aromadendrene	1.33	Terpinen-4-ol	7.57–10.51
β-eudesmol	0.46	eugenol + α-cubebene	<0.01–0.04	allo-aromadendrene	0.55	α-terpineol	1.00–1.74
				δ-cadinene	1.18	c-dihydro-carvone	0.19–0.22
				globulol	0.29	linalyl acetate	0.46–2.28
				terpinolene	3.62	β-caryophyllene	0.01–0.61
						6-methyl-5-heptene-2-one	<0.01–0.04
						t-α-bergamotte	<0.01–1.71
						*m*-thymol	<0.01–0.83
						citronellol + nerol	<0.01–0.07

* gas chromatography analysis performed on GC-FID-MS Perkin Elmer Clarus 500 using column DB5-MS. 60 m × 0.25 mm × 0.25 µm.

**Table 2:** Minimum inhibitory and bactericidal concentration (MIC/MBC) [mg/mL] of cajeput, lemongrass, tea tree, and thyme essential oils against 27 uropathogenic strains in artificial urine, determined by broth microdilution assay. Median values from three independent measurements are shown.

Bacterial Strain ^1^	Antibiotic Resistance	Essential Oils							
		Cajeput		Lemongrass		Tea Tree		Thyme	
		MIC	MBC	MIC	MBC	MIC	MBC	MIC	MBC
Ec ATCC 25922		12.5	12.5	25	25	1.56	1.56	1.56	1.56
Ec CHD1		3.13	3.13	1.56	3.13	1.56	1.56	0.78	1.56
Ec CHD3		3.13	3.13	0.63	6.25	1.56	1.56	0.78	0.78
Ec CHD22		3.13	3.13	25	25	1.56	1.56	1.56	1.56
Ec CHD94		6.25	6.25	25	25	1.56	1.56	1.56	1.56
Ec UTI89		6.25	6.25	25	25	1.56	1.56	0.78	0.78
Ec CHD16	*bla*_CTX-M-15_	6.25	12.5	12.5	25	3.13	6.25	3.13	3.13
Ec IR3	*bla*_CTX-M-15_, *bla*_TEM-1B_	3.13	6.25	1.56	1.56	3.13	3.13	1.56	3.13
Ec H75	*bla*_NDM-1_	12.5	12.5	25	25	3.13	3.13	3.13	6.25
Ec 1949820	fluoroquinolone	25	50	50	50	3.13	0.78	3.13	3.13
Ec CDF2	*mcr-1*	1.56	1.56	1.56	1.56	1.56	1.17	0.78	0.78
Kp 595		3.13	3.13	3.13	3.13	1.56	1.56	1.56	1.56
Kp CHD67		3.13	3.13	3.13	6.25	1.56	1.56	1.56	1.56
Kp CHD99		25	50	50	50	3.13	3.13	25	50
Kp ATCC BAA1705	*bla*_KPC_	25	50	25	25	3.13	3.13	6.25	12.5
Kp ATCC BAA2146	*bla*_NDM-1_	12.5	50	25	50	6.25	6.25	25	50
Ecl CHD57		25	50	25	25	1.56	3.13	1.56	1.56
Ecl CHD60		3.13	6.25	6.25	12.5	0.78	1.56	0.78	0.78
Pm CHD72		25	50	25	25	6.25	6.25	25	25
Pm CHD76		50	50	12.5	25	6.25	6.25	25	25
Pa ATCC 27853		50	50	25	50	12.5	50	25	50
Pa CHD80		25	50	25	50	25	25	25	50
Pa CHD81		50	50	25	50	50	50	50	50
Ef CHD30		6.25	6.25	3.13	1.56	12.5	12.5	6.25	6.25
Ef CHD31		12.5	12.5	1.56	1.56	12.5	12.5	6.25	12.5
Ss Ho94		1.56	1.56	0.78	0.78	1.56	1.56	1.56	1.56

^1^ Ec—Escherichia coli, Kp—Klebsiella pneumoniae, Ecl—Enterobacter cloacae, Pm—Proteus mirabilis, Pa—Pseudomonas aeruginosa, Ef—Enterococcus faecalis, Ss—Staphylococcus saprophyticus.

**Table 3:** Minimum inhibitory and bactericidal concentration (MIC/MBC) against four *E. coli* strains ^$^ in artificial urine of tea tree and thyme essential oils, fosfomycin, nitrofurantoin, and pivmecillinam, alone and fold, change MIC/MBC reduction in combination with tea tree or thyme essential oil. Median values from three independent measurements are shown.

Essential Oil/Antibiotic	MIC	Fold Change MIC Reduction in Combination with *:		MBC	Fold Change MBC Reduction in Combination with *:	
		Tea Tree	Thyme		Tea Tree	Thyme
thyme	3.13 mg/mL	8 ^#^ (0.531)	-	3.13 mg/mL	8 ^#^ (0.547)	-
tea tree	1.56 mg/mL	-	8 ^#^ (0.547)	3.13 mg/mL	-	8 ^#^ (0.547)
nitrofurantoin	8 µg/mL	2 (1)	2 ^§^ (0.75)	8 µg/mL	2 ^§^ (1)	1 (1.25)
fosfomycin	32 µg/mL	2 (0.625)	4 ^§^ (0.75)	64 µg/mL	4 (0.75)	2 (0.75)
pivmecillinam	2 µg/mL	4 ^§^ (0.75)	2 ^§^ (0.75)	8 µg/mL	8 ^#^ (0.625)	4 ^§^ (0.688)

^$^*E. coli* CHD3, *E. coli* CHD22, *E. coli* CHD94, and *E. coli* UTI89; * fractional inhibitory/bactericidal concentrations indices (FICI/FBCI) indicated in brackets; # *p* < 0.01; § *p* < 0.05.
